# Microbiological Contamination of Drinking Water Associated with Subsequent Child Diarrhea

**DOI:** 10.4269/ajtmh.15-0274

**Published:** 2015-11-04

**Authors:** Stephen P. Luby, Amal K. Halder, Tarique Md. Huda, Leanne Unicomb, M. Sirajul Islam, Benjamin F. Arnold, Richard B. Johnston

**Affiliations:** International Centre for Diarrhoeal Disease Research, Bangladesh (icddr,b), Dhaka, Bangladesh; Stanford University, Stanford, California; London School of Hygiene and Tropical Medicine, London, United Kingdom; School of Public Health, University of California-Berkeley, Berkeley, California; Water and Environmental Sanitation Section, United Nations Children's Fund (UNICEF) Bangladesh, Dhaka, Bangladesh; Department of Public Health, Environmental and Social Determinants of Health, World Health Organization, Geneva, Switzerland

## Abstract

We used a prospective, longitudinal cohort enrolled as part of a program evaluation to assess the relationship between drinking water microbiological quality and child diarrhea. We included 50 villages across rural Bangladesh. Within each village field-workers enrolled a systematic random sample of 10 households with a child under the age of 3 years. Community monitors visited households monthly and recorded whether children under the age of 5 years had diarrhea in the preceding 2 days. Every 3 months, a research assistant visited the household and requested a water sample from the source or container used to provide drinking water to the child. Laboratory technicians measured the concentration of *Escherichia coli* in the water samples using membrane filtration. Of drinking water samples, 59% (2,273/3,833) were contaminated with *E. coli*. Of 12,192 monthly follow-up visits over 2 years, mothers reported that their child had diarrhea in the preceding 2 days in 1,156 (9.5%) visits. In a multivariable general linear model, the log_10_ of *E. coli* contamination of the preceding drinking water sample was associated with an increased prevalence of child diarrhea (prevalence ratio = 1.14, 95% CI = 1.05, 1.23). These data provide further evidence of the health benefits of improved microbiological quality of drinking water.

## Introduction

The contribution of drinking water contaminated with human feces to the global burden of child diarrhea remains uncertain.^1^,^2^ Communities whose water is contaminated with human feces generally have multiple sources of environmental fecal contamination,^3^ making it difficult to disentangle the marginal impact of contaminated water.

Measuring fecal contamination in water is difficult. Traditional measures use organisms commonly found in human feces, but these indicator organisms are only weakly associated with the presence of pathogens.^4^ Moreover, measurements of fecal indicator organisms in water are highly variable.^5^ This high variability biases studies evaluating relationships between water quality and diarrhea toward the null.^6^,^7^ In addition, when water samples are collected simultaneously with disease information, there is a risk of bias due to reverse causation either due to actions by the household to clean water in response to illness, or to excess fecal shedding by an ill child that alters the concentration of fecal contamination in drinking water.

The objective of this study was to use a large set of repeated, prospective measures of water quality and child diarrhea collected within an evaluation of a water, sanitation, and hygiene intervention project^8^ to estimate the relationship between water quality and subsequent diarrhea among children < 5 years of age.

## Methods

### Study population.

The evaluation of the Sanitation, Hygiene Education and Water Supply in Bangladesh (SHEWA-B) program including steps to protect human subjects has been previously described.^8^,^9^ In brief, the SHEWA-B program targeted 68 subdistricts (upazilas) in 19 districts across Bangladesh. Upazilas are further subdivided into unions. We listed all of the unions and their populations in the 68 targeted upazilas and randomly selected 50 unions with the probability of selection proportional to the size of the union. For each SHEWA-B intervention upazila where a union was chosen for evaluation, we selected a control upazila that had similar geography, hydrogeology, infrastructure, agricultural productivity, and household construction and where government collaborators confirmed that no other major water, sanitation, and hygiene programs were operating. We selected unions for evaluation in the control upazilas using the same probability of selection proportional to size used to select unions for evaluation in the intervention upazilas. Because the SHEWA-B intervention included some activities aimed at improving drinking water quality, this analysis was restricted to the 50 control communities.

As no village level census was available, within each selected union we listed all village names and used a random number generator to select the evaluation village. Field-workers approached the households closest to the village center and sought consent for an interview if they had a child < 5 years of age. To enroll the next household, field-workers looked for the next closest household with a child < 5 years of age. The first 10 enrolled households who had at least one child < 3 years of age at the initial interview, and so would remain < 5 years of age during 2 years of follow-up, were also invited to participate in monthly disease surveillance and quarterly water testing (*N* = 500) beginning in September 2007.

### Monthly surveillance.

Field-workers recruited a female community resident ≥ 18 years of age, who had completed at least 8 years of formal education as a community monitor. The community monitor visited the enrolled households monthly and administered a brief questionnaire to collect information on each child < 5 years of age. One of the monthly surveillance questions asked whether the child had diarrhea (≥ 3 loose stools within 24 hours) during the preceding 2 days. This surveillance continued for 24 months.

### Household wealth.

We used the principal component analysis of 21 household possessions and construction characteristics to evaluate household wealth,^8^,^10^ and the first principal component as the wealth score.^11^

### Water collection and laboratory processing.

Every 3 months field-workers requested the caregiver of the youngest child in the household to draw a glass of water as if her child wanted a drink. Field-workers transferred the drinking water into a sterile plastic bottle, placed it in a box with ice packs and brought it to icddr,b's Environmental Microbiology Laboratory.

Within 24 hours of collection laboratory technicians filtered 100 mL of the collected drinking water sample through separate Millipore membrane filters, placed the filter papers on modified thermotolerant *Escherichia coli* (mTEC) agar media (Difco^™^; Becton, Dickinson and Company, Franklin Lakes, NJ), and incubated the plates at 35°C for 2 hours and then at 44.5°C for another 22 hours. Laboratory technicians counted red or magenta colonies as *E. coli*.^12^ When the result were too numerous to count, technicians either inoculated 100 μL of water directly on mTEC agar media using the drop plate technique or diluted a 10 mL of the original sample with 90 mL sterile water and filtered the specimen through the Millipore filters. The diluted specimens were incubated as above. Plates with non-confluent colonies were used to count colonies. Counts from dilute plates were multiplied by the magnitude of dilution to generate counts per 100 mL.

Technicians periodically tested water samples spiked with *E. coli* (ATCC 25922) strain as a positive control and sterile water as a negative control.

### Statistical analysis.

We considered water samples with < 1 *E. coli*/100 mL to be uncontaminated and samples with ≥ 1 *E. coli* to be contaminated. We converted the *E. coli* concentrations to their base 10 logarithm for calculating geometric means, which we report as the geometric mean *E. coli* concentration among the subset of contaminated samples.

Regression modeling requires a nonzero value to be used when the *E. coli* concentration is < 1/100 mL or the observations cannot be included in the model. For regression modeling, we replaced *E. coli* < 1/100 mL with 0.5 (half the limit of detection).

We included observations from households that had water quality measurements and one or more measurement of diarrhea morbidity 3–100 days after collection of the water sample. We ignored diarrhea measurements < 3 days after water quality measurement because this is shorter than the incubation period of most enteric pathogens. We ignored measurements > 100 days after water quality measurement so that missing water measurements would not result in earlier water quality measures predicting diarrhea over quite different time frames. If multiple measurements of water quality were available 3–100 days before diarrhea morbidity was assessed, we used the water quality measurement closest to the assessment of diarrhea for the predictive modeling.

To evaluate the association between the exposure variables—drinking water quality, child and household characteristics—and diarrhea, we estimated prevalence ratios using a log-binomial regression model.^13^ To account for the repeated observations for diarrhea in single household and the clustering of observations in villages, we used a robust sandwich standard error estimator clustered at the village level to calculate 95% confidence intervals. To assess if the association between *E. coli* concentration in drinking water and diarrhea was independent of other exposures associated with diarrhea, we constructed a multivariable model. We began with the bivariate model of the association between *E. coli* concentration and diarrhea and added child- or household-level characteristics that were associated (*P* < 0.05) with diarrhea in bivariate analyses.

To evaluate the impact of the elapsed time between the water collection and the diarrhea measurement on the association between the presence of *E. coli* and diarrhea, we divided the distribution of elapsed times by quartiles and by the median and explored differences in the prevalence ratios between presence of *E. coli* and diarrhea across different elapsed sampling intervals.

To estimate the population attributable fraction of diarrhea due to *E. coli* contamination, we subtracted the diarrhea prevalence among all included children by the diarrhea prevalence in the subgroup of those children whose prior household water quality measurement was < 1 *E. coli*/100 mL and divided this difference by the diarrhea prevalence of all included children.^14^

## Results

Among the 500 enrolled control households who completed the baseline survey and agreed to participate, the field team collected at least one measure of household drinking water quality and 2 months of diarrhea surveillance information for 497 households. These 497 households had a mean of 1.3 children under the age of 5 years (Table 1). The most common drinking water source was a shallow tubewell (79%). Of households, 51% owned a toilet or latrine, though 93% reported access to a toilet or latrine.

**Figure 1.**
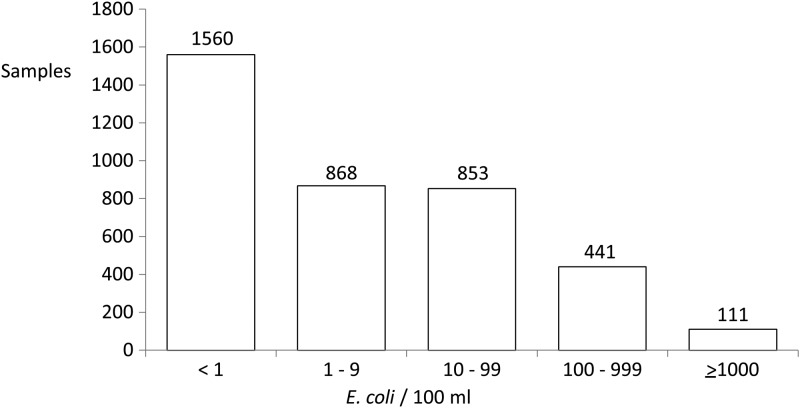
*Escherichia coli* concentration among all drinking water samples (*N* = 3,833).

**Figure 2.**
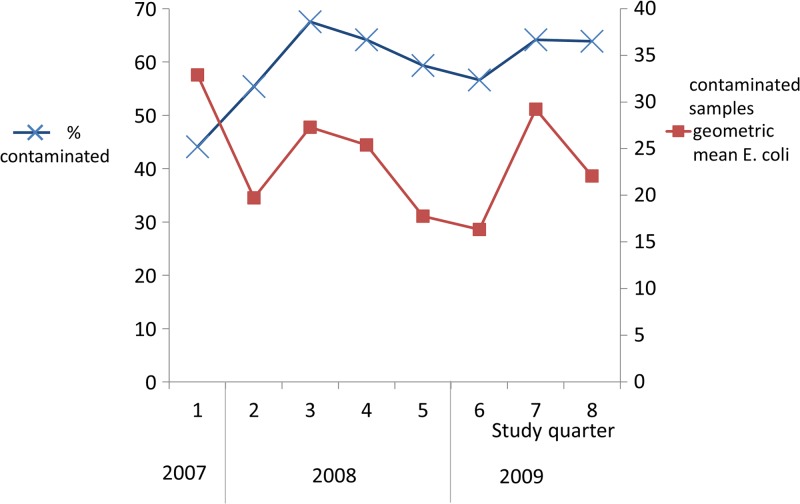
Proportion of drinking water samples contaminated with *Escherichia coli* and geometric mean *E. coli* concentrations in contaminated samples over time (*N* = 3,833).

Among these 497 households, 408 (83%) provided eight drinking water samples, 62 (12%) provided seven, and 27 (5%) provided one to six samples over the 24 months of surveillance. Of collected samples, 59% (2,273/3,833) were contaminated with *E. coli*, with a geometric mean of 23 colony forming units (CFU) *E. coli*/100 mL (95% CI = 21, 25) among the contaminated samples. Assuming that the samples with no detectable *E. coli* had 0.5 *E. coli*/100 mL, the geometric mean of all water samples was 5 CFU *E. coli*/100 mL. Of collected samples, 14% (552) had ≥ 100 CFU *E. coli*/100 mL (Figure 1

). The proportion of contaminated samples increased after the first two quarters (Figure 2

).

**Figure 3.**
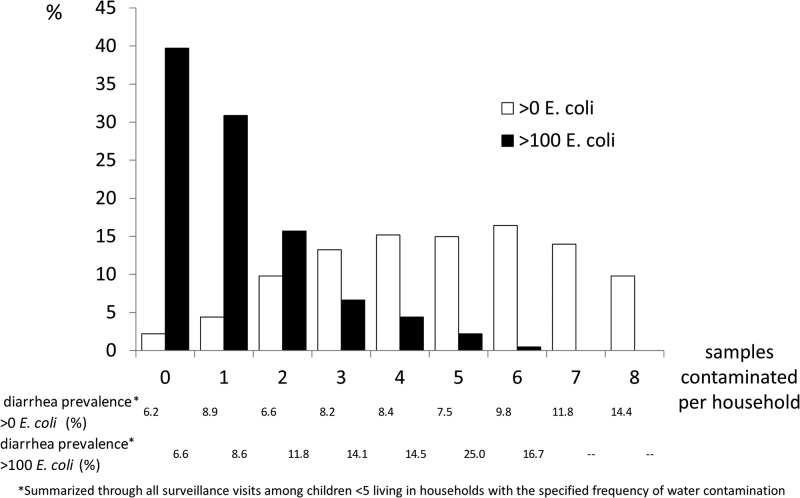
The percent of samples contaminated with *Escherichia coli* per household among household that provided a sample for each of the eight quarters of the study (*N* = 408).

Most households' drinking water was intermittently contaminated. Among the 408 households who contributed a water sample during each of the eight quarters of the study, only nine (2%) had drinking water with no *E. coli* contamination from any of the eight water samples and 40 (10%) had *E. coli* contamination detected in each water sample (Figure 3

). Over half of these households (60%) had at least one drinking water sample with > 100 *E. coli*/100 mL and 29% had more than one (Figure 3).

Community monitors enrolled 544 children in the first quarter of surveillance. During 24 months of follow-up, 79 children were born into and 11 children moved into these households: 1 child aged out, 5 children died, and 10 children moved or dropped out. Among the 14,094 potential monthly child assessments, community monitors collected data for 13,918 (99%). For 12,193 monthly child assessments (88%) microbiologists collected and analyzed a drinking water sample within the preceding 100 days.

In the 12,193 monthly follow-up visits 4–100 days after drinking water sample collection, mothers reported that their child had diarrhea in the preceding 2 days in 1,161 (9.5%). Children whose previous household drinking water samples had *E. coli* contamination were 22% more likely to have diarrhea at the next follow-up visit compared with children whose household drinking water samples had no detectable *E. coli* (Table 2). Children whose parents had formal education and children living in wealthier households had less diarrhea (Table 2). Children under 2 years of age were more likely to have diarrhea than older children. Diarrhea was more common during the first year of surveillance than during the second.

The strength of association between *E. coli* contamination in drinking water and child diarrhea varied by the elapsed time between drinking water sample collection and diarrhea history (Table 3). Children whose household drinking water samples were collected 3–46 days previously and were contaminated with *E. coli* were 35% more likely to have diarrhea than children whose household drinking water samples collected from that same time frame had no detectable *E. coli* (Table 3). *Escherichia coli* contamination of drinking water samples collected > 46 days before interview were not significantly associated with child diarrhea.

The strength of association between *E. coli* contamination in drinking water and child diarrhea varied by child age. Children aged 6 to < 12 months had the highest prevalence of diarrhea (14.8%) and the strongest association between *E. coli* contamination in drinking water and diarrhea (Table 3).

Children whose household drinking water samples were contaminated with progressively higher concentration of *E. coli*, up to 999 *E. coli*/100 mL, had higher diarrhea prevalence, though only between 100 and 999 *E. coli*/100 mL were the differences large enough to confidently exclude chance (Table 4). Fewer samples were contaminated with ≥ 1,000 *E. coli*/100 mL, and children in these households had somewhat lower diarrhea prevalence compared with children whose household water had 100–999 *E. coli*/100 mL. Restricting the analysis to assessments 3–46 days after water sample collection generated similar findings, though the prevalence ratios were larger (Table 4).

The strength of the association between the log of *E. coli* contamination of drinking water and subsequent child diarrhea was unchanged in the multivariable analysis that included potential confounders. Each 10-fold increase in *E. coli* contamination in drinking water was associated with a 14% increase in diarrhea in the subsequent visit (Table 5). Restricting the analysis to diarrhea assessments 3–46 days after water sample collection slightly strengthened this association (Table 5).

In the analysis restricted to assessments 3–46 days after water sample collection, substituting drinking water contamination (dichotomous) for log_10_ *E. coli* as the measure of drinking water quality, *E. coli* contamination increased the risk of subsequent diarrhea by 35% in the bivariate analysis and 34% in the multivariable analysis (Table 5).

Children living in households where water was more consistently contaminated with *E. coli* had a higher prevalence of diarrhea than children living in households where water was infrequently contaminated (Figure 3). Children living in households where water was commonly contaminated with > 100 *E. coli*/100 mL were much more likely to have diarrhea than children living in households where *E. coli* contamination was at a lower concentration or where high level of contamination was less common (Figure 3).

The proportion of children with diarrhea in the 3–46 days after water collection was 9.39%. The prevalence of diarrhea among those children whose previous household water samples had < 1 *E. coli*/100 mL was 7.79%. The population attributable fraction of diarrhea attributed to contaminated water in the 3–46 days after water collection was 17%.

## Discussion

Drinking water in rural Bangladesh was commonly contaminated with bacteria indicating fecal contamination. This contamination was usually low level and varied between households and over time consistent with prior reports from rural Bangladesh.^16^–^18^ The level of *E. coli* contamination in drinking water was associated with subsequent diarrhea, with little evidence of confounding. The contribution of *E. coli* contamination to the overall burden of diarrhea was modest at 17% based on the population attributable fraction. A causal relationship between fecal contamination of drinking water and child diarrhea is supported by the measurement of water quality prior to the outcome measurement, the relationship between increasing *E. coli* concentration in drinking water and increasing risk of diarrhea, the stronger association with reported diarrhea in the period soon after the water sample was collected (3–46 days) than in the later period after sample collection (47–100 days), the strongest association between water contamination and diarrhea at the age when children are most immunologically susceptible, and the stability of the relationship when accounting for potential confounding exposures. A recent randomized intervention trial of improved drinking water microbiological quality and reduced reported diarrhea among residents of rural Bangladesh that used shallow tubewells for drinking water, in settings very similar to this reported in SHEWA-B evaluation, provides further evidence of causality.^19^

Prior studies evaluating the relationship between indicators of fecal contamination in drinking water and subsequent diarrhea have produced conflicting results.^20^ Some authors reported no association between microbial indicators of water quality and diarrhea,^2^,^21^,^22^ whereas some reported a modest association.^23^–^26^ Five circumstances could affect the measured association between indicator organisms and diarrhea. First, indicator organisms are only weakly correlated with the presence of enteric pathogens. Indeed, many authors report no association between indicator organisms and enteropathogens, though studies with larger sample sizes are more likely to detect an association.^4^ Thus, even if there is a strong relationship between enteric pathogens in drinking water and diarrhea, the weak correlation between fecal indicator bacteria and pathogens would weaken a measured association between fecal indicator bacteria and diarrhea.

Second, population immunity to common circulating enteropathogens would weaken the association between bacterial indicators and diarrhea. Immunologically susceptible populations experience high attack rates and a strong association between dose of enteropathogen and risk of diarrhea.^27^,^28^ Widespread immunity among community residents to pathogens commonly present in drinking water attenuates a simple relationship between dose of exposure and risk of disease.^29^,^30^ Our finding that the strongest association between *E. coli* contamination in drinking water and diarrhea was among children aged 6–12 months, when maternally acquired immunoglobulin is waning and the adaptive immune system is less developed,^31^,^32^ is consistent with acquired immunity mediating the relationship between water contamination and diarrhea.

Third, high variability in the measurements of drinking water microbial contamination would weaken their association with diarrhea. *Escherichia coli* levels in study households varied substantially among visits (Figure 3). In other contexts, microbial indicators of drinking water quality have varied markedly from day to day and from hour to hour.^5^ In the settings of high variability, a single measurement of microbial drinking water will be a poor predictor of health outcome weeks or months later because of misclassification of the exposure and the resulting regression dilution bias.^6^

Fourth, intervention studies to alter microbial quality of drinking water risk bias because people who receive an intervention to improve drinking water quality may, out of courtesy, underreport diarrhea.^1^ Because the participants in this study received no intervention and were not informed of the water quality measurements, this bias cannot explain the observed associations.

Finally, the presence of a child with diarrhea in the household might increase the risk of household fecal contamination through maternal hands that clean up the child and also fetch water or might alter water treatment or storage practices. Studies that assess drinking water quality at the same time when they collect data on diarrhea may find a relationship between diarrhea and drinking water quality, but the direction of causality may move from the child's diarrhea to drinking water.^22^ Because this study collected drinking water on a separate visit that was at least 3 days prior to the visit collecting diarrhea information, there is no risk of this bias altering the association.

Altogether, the three factors that would be expected to weaken the association between microbial indicators of water contamination and diarrhea—the weak association between indicator organisms and pathogens, population immunity to pathogens, and the high variability of measurements of water quality of diarrhea—were all present within this study and likely weakened the association between measures of drinking water quality and diarrhea. By contrast, the two conditions that might artificially strengthen an association between drinking water quality and diarrhea, courtesy bias and reverse causality, were not factors in this study. Thus, within this context, the measured association likely represents a minimal estimate of the contribution of drinking water quality to diarrhea.

In this study, the risk of diarrhea increased even with moderate increases in *E. coli* contamination. Indeed, between *E. coli* concentrations < 1 and 1,000/100 mL, the results suggest a dose–response effect. This contrasts with an influential earlier study using a smaller number of observations (1,062 water samples tested) from the urban Philippines that found no increased risk of diarrhea until a threshold > 1,000 *E. coli* (or other indicator organism)/100 mL was met.^23^ Our data demonstrate that the threshold effect observed in urban Philippines is not a universal phenomenon.

There are important limitations to this analysis. This study was conducted within a context of modest but frequent contamination of groundwater.^17^,^18^ It is possible that microbial indicators of drinking water quality are differentially informative of diarrhea risk in other contexts. However, these observations do support the notion that dose of exposure of fecal organisms in drinking water increases the risk of diarrhea and that, at least within the context of a high water table and high population density that is typical of rural Bangladesh, the amount of exposure to fecal contamination in drinking water contributes meaningfully to the risk of child diarrhea.

Although the study hypothesis was prespecified in the program evaluation, the analytical plan was not. There is some risk that the group chosen for analysis (controls but not intervention households) and the cut points used for bivariate analysis affected the observed statistical associations. However, restricting the study population to controls provided the most direct evaluation of the hypothesis, and inclusion of the intervention group did not alter any of the principal findings (data not shown).

Overall, the conclusions were robust to categorical or continuous definitions of *E. coli* concentrations. Although the stratified analysis did not show a significant association at each categorical range of *E. coli* contamination with diarrhea, the pattern of stratum-specific prevalence ratios suggests a trend of increasing risk with higher contamination. Moreover, the log concentration of *E. coli* used in the multivariate model provides a more robust assessment of the dose–response relationship. It does not depend on arbitrary cut points that stratified analysis requires but, instead, assesses the relationship throughout the data.

Fecal indicator measurements in drinking water remain an imperfect measure of health risk, but this study provides further evidence of the health benefits of improved microbiological quality of drinking water. Continued efforts to improve microbiological quality of drinking water have the potential to reduce child diarrhea in low-income countries.

## Figures and Tables

**Table 1:** Characteristics of participating households, rural Bangladesh 2007

Characteristic	All participating households (*N* = 497)	
General	(*n*)	
Number household residents	2,676	5.4
Number of children age < 5 years	654	1.3
Father of the youngest child lacked formal education	172	35%
Mother of the youngest child lacked formal education	136	27%
Occupation of father of the youngest child†	(*n*)	(%)
Laborer	119	24
Farmer/rickshaw puller or homemaker	164	33
Skilled worker	37	7
Working abroad	39	8
Salaried employee	48	10
Business owner	81	16
Drinking water source	(*n*)	(%)
Shallow tubewell	395	79
Deep tubewell	45	9
Tara pump	23	5
Piped water	13	3
Protected well	11	2
Surface water	6	1
Other	4	1
Owned source of drinking water	136	27
Owned a latrine or toilet	251	51
Owned an improved latrine*	198	40
Had access to a toilet or latrine	464	93
Proportion who owned	(*n*)	(%)
House†	462	93
Wardrobe†	146	29
Bicycle†	134	30
Mobile phone†	156	31
Black and white television†	90	18
Color television†	53	11
Sewing machine†	36	7
Refrigerator	14	3
Motor cycle	8	2
Mean number of items owned	(*n*)	(Mean)
Tables†	497	1.0
Chairs†	497	2.2
Watches/clocks†	497	1.4
Beds†	497	0.9
Inexpensive sleeping cots†	497	1.2
Acres of agricultural land†	497	0.93
Acres of non-agricultural land†	497	0.20
House construction	(*n*)	
Tin roof†	446	90%
Cement floor†	43	9%
Brick walls†	45	9%
Mean number of rooms†	497	2.2
Household electrical connection†	247	50%
Cooking fuel†	(*n*)	(%)
Crop residue/grass	288	58
Wood	123	25
Dung	85	17

* Following World Health Organization and UNICEF definitions.^15^

† Items used to construct the wealth index.

**Table 2:** Bivariate relationship between household and child characteristics and exposures with subsequent diarrhea (3–100 days) among children under age of 5 years

Exposures	Number of monthly observations	No. (%) of monthly visits with this exposure	No. (%) of monthly visits with diarrhea		Prevalence ratio	95% confidence interval*	*P* value*
			With exposure	Without exposure			
Drinking water							
Previous drinking water sample†							
Any *Escherichia coli* contamination	12,192	7,199 (59)	741 (10.3)	420 (8.4)	1.22	1.00, 1.50	0.05
Log_10_ *E. coli* contamination	12,192	–	–	–	1.15	1.05, 1.26	0.003
Drinking water source	12,192						
Shallow tubewell		9,557 (78)	1,939 (9.2)	–	–	–	–
Deep tubewell		1,160 (10)	231 (12.2)	1,939 (9.2)	1.33	0.90, 1.98	0.15
Tara pump		598 (5)	148 (11.2)	1,939 (9.2)	1.22	0.67, 2.21	0.51
Other		877 (7)	99 (8.6)	1,939 (9.2)	0.93	0.28, 3.14	0.91
Household characteristics							
Mother's education > 0 years	12,192	8,814 (72)	819 (9.3)	342 (10.1)	0.92	0.73, 1.15	0.45
Each year of mother's education	12,192	–	–	–	0.963	0.933, 0.993	0.02
Father's education > 0 years	12,129‡	7,899 (65)	688 (8.7)	468 (11.1)	0.79	0.59, 1.06	0.11
Each year of father's education	12,129	–	–	–	0.979	0.950, 1.009	0.17
Owned radio	12,192	2,776 (23)	221 (8.0)	940 (10.0)	0.80	0.58, 1.11	0.17
Owned television	12,192	3,385 (28)	270 (8.0)	891 (10.1)	0.79	0.58, 1.07	0.13
Owned mobile phone	12,192	2,768 (23)	233 (8.4)	928 (9.9)	0.85	0.63, 1.16	0.31
Had electricity	12,192	6,207 (51)	541 (8.7)	620 (10.4)	0.84	0.62, 1.13	0.26
Owned source of drinking water	12,192	3,482 (29)	309 (8.9)	852 (9.8)	0.91	0.70, 1.17	0.45
Owned toilet	12,192	6,247 (54)	632 (10.1)	529 (8.9)	1.14	0.89, 1.46	0.31
Used improved latrine	12,192	2,894 (24)	234 (8.1)	927 (10.1)	0.81	0.59, 1.12	0.21
Wealth index quintile	12,192						
1–reference (poorest)		2,286 (18)	252 (11.0)	–	–	–	–
2		2,137 (20)	229 (10.7)	252 (11.0)	0.97	0.69, 1.37	0.87
3		2,442 (18)	251 (10.3)	252 (11.0)	0.93	0.70, 1.24	0.63
4		2,778 (21)	220 (7.9)	252 (11.0)	0.72	0.50, 1.02	0.07
5–richest		2,549 (23)	209 (8.2)	252 (11.0)	0.74	0.55, 1.01	0.06
Wealth index continuous	12,192	–	–	–	0.87	0.77, 0.99	0.04
Child characteristic							
Male child	12,192	6,055 (50)	580 (9.6)	581 (9.5)	1.01	0.84, 1.22	0.90
Age < 2 years	12,192	4,304 (35)	504 (11.7)	657 (8.3)	1.41	1.17, 1.69	< 0.001
Year 1 surveillance (vs. year 2)	12,192	5,220 (43)	621 (11.9)	540 (7.8)	1.54	1.21, 1.95	< 0.001
Month since initiation of surveillance	12,192	–	–	–	0.968	0.950, 0.986	0.001
Exclusive breast-feeding last 24 hours (among children age < 2 years)	3,737	319 (9)	29 (9.1)	438 (12.8)	0.71	0.46, 1.09	0.12

* Accounting for clustering at the village level using a robust sandwich standard error estimator.

† Collected 3–100 days before child health questionnaire.

‡ Father's education was not reported from three households.

**Table 3:** Association between *Escherichia coli* contamination in drinking water and diarrhea by time between sample collection and reported diarrhea and by age subgroups

Subgroups	Number of observation	No. (%) monthly visits with *E. coli* ≥ 1/100 mL	No. (%) monthly visits with *E. coli* ≥ 1/100 mL and diarrhea	Prevalence ratio*	95% confidence interval†	*P* value†
Quartiles of days between water quality and diarrhea measurement						
3–23	2,884	1,758 (61)	183 (10.4)	1.23	0.92, 1.66	0.17
24–46	3,046	1,746 (57)	185 (10.6)	1.47	1.13, 1.90	0.004
47–68	3,040	1,764 (58)	174 (9.9)	1.07	0.78, 1.46	0.68
69–100	3,222	1,931 (60)	199 (10.3)	1.16	0.89, 1.55	0.25
Days between water quality and diarrhea measurement by the median						
3–46	5,930	3,504 (59)	368 (10.5)	1.35	1.07, 1.70	0.012
47–100	6,262	3,695 (59)	373 (10.1)	1.12	0.89, 1.41	0.321
Child age in months						
< 6	515	299 (58)	29 (9.7)	0.95	0.46, 1.97	0.895
6 to < 12	862	520 (60)	91 (17.5)	1.62	1.12, 2.32	0.009
12 to < 18	1,284	737 (57)	88 (11.9)	1.17	0.74, 1.84	0.506
18 to < 24	1,644	906 (55)	102 (11.3)	1.05	0.73, 1.51	0.784
24 to < 60	7,899	4,738 (60)	431 (9.1)	1.26	1.00, 1.60	0.048

* The prevalence of diarrhea in households with *E. coli* ≥ 1/100 mL/prevalence of diarrhea in households with *E. coli* < 1/100 mL.

† Accounting for clustering at the village level using a robust sandwich standard error estimator.

**Table 4:** Diarrhea prevalence by level of water contamination

*Escherichia coli* concentration/100 mL drinking water	No. of diarrhea measurements	Prevalence (%) of diarrhea in subsequent evaluation	Prevalence ratio (95% confidence interval)
Reported diarrhea 3–100 days after water sample collection (*N* = 24,334)			
< 1	4,993	8.4	Reference
1–9	2,751	8.6	1.02 (0.83, 1.26)
10–99	2,700	9.9	1.17 (0.92, 1.49)
100–999	1,382	14.1	1.68 (1.30, 2.17)
≥ 1,000	366	11.7	1.39 (0.93, 2.10)
Reported diarrhea 3–46 days after water sample collection (*N* = 12,103)			
< 1	2,426	7.8	Reference
1–9	1,345	9.4	1.20 (0.95, 1.52)
10–99	1,286	9.3	1.19 (0.89, 1.62)
100–999	696	14.1	1.81 (1.35, 2.41)
≥ 1,000	177	13.6	1.74 (1.09, 2.78)

**Table 5:** Multivariable analysis of household and child characteristics and exposures with subsequent diarrhea among children under 5 years of age (*N* = 12,192)

Characteristic	Bivariate prevalence ratio (95% confidence limit)	Multivariable prevalence ratio* (95% confidence limit)	*P* value†
Reported diarrhea 3–100 days after water sample collection (*N* = 12,192)			
Log_10_ *Escherichia coli* contamination	1.15 (1.05, 1.26)	1.14 (1.05, 1.23)	0.003
Child age in months	0.987 (0.979, 0.995)	0.992 (0.985, 0.999)	0.023
Month since initiation of surveillance	0.968 (0.950, 0.986)	0.973 (0.957, 0.989)	0.001
Each year of mother's education	0.963 (0.933, 0.993)	0.974 (0.943, 1.006)	0.111
Wealth index	0.875 (0.771, 0.992)	0.931 (0.813, 1.067)	0.306
Reported diarrhea 3–46 days after water sample collection (*N* = 5,930)			
Log_10_ *E. coli* contamination	1.18 (1.06, 1.30)	1.16 (1.06, 1.27)	0.001
Child age in months	0.990 (0.980, 0.999)	0.994 (0.985, 1.002)	0.153
Month since initiation of surveillance	0.976 (0.957, 0.995)	0.979 (0.963, 0.995)	0.011
Each year of mother's education	0.966 (0.935, 0.998)	0.982 (0.947, 1.02)	0.351
Wealth index	0.857 (0.735, 0.999)	0.905 (0.756, 1.084)	0.278
Reported diarrhea 3–46 days after water sample collection (*N* = 5,930)			
*E. coli* ≥ 1	1.35 (1.07, 1.70)	1.34 (1.08, 1.65)	0.007
Child age in months	0.990 (0.980, 0.999)	0.993 (0.984, 1.002)	0.136
Month since initiation of surveillance	0.976 (0.957, 0.995)	0.979 (0.963, 0.995)	0.012
Each year of mother's education	0.966 (0.935, 0.998)	0.981 (0.945, 1.020)	0.336
Wealth index	0.857 (0.735, 0.999)	0.902 (0.754, 1.080)	0.261

* The prevalence ratio was calculated using general linear modeling; standard errors were calculated using robust sandwich variance estimates to account for village level clustering and repeated household sampling.

† For the multivariable analysis.
